# Meetings of Societies

**Published:** 1926

**Authors:** 


### Meetings of Societies.

### Bristol Medico-Chirurgical Society.

THE Annual Meeting was held in the Society's room at the University on Wednesday, October 13th. The President, Mr. T. Carwardine, proposed from the chair the name of Dr. P. Watson-Williams for election to the Honorary Membership. He knew that he need not remind the Society how great was the debt of gratitude they owed the candidate. His editorship of the *Journal* had embraced the very difficult period of the war and the succeeding years. Those who knew the inner history of the Society realised that it was owing entirely to the personal devotion of the Editor that the *Journal* had been able to survive those difficult times, and were cognisant of the great amount of time and energy devoted to their service. The proposal was carried by acclamation, and Dr. P. Watson-Williams was elected an Honorary Member of the Society. Mr. Carwardine then vacated the chair in favour of Dr. A. L. Flemming, the incoming President.

Dr. Flemming delivered his Presidential Address on "**Anæsthesia,**" to be published in the next issue.

Dr. P. Watson-Williams having entered, the President said that he regarded it as particularly grateful that his first official duty was to inform Dr. Watson-Williams of his election to Honorary Membership, and to welcome him in the name of the Society. This represented the highest honour the Society could do one of their number. It was reserved for those only to whom the Society could make no other gesture of recognition, and that it was sparingly bestowed was evidenced by there being now only four Honorary Members. He hoped that Dr. Watson-Williams would long enjoy his Honorary Membership.

Dr. P. Watson-Williams thanked the Society for the honour they had done him.

The Secretaries' report and balance sheets were presented and accepted, and the following officers were elected :—

*President-Elect*—Dr. R. S. S. Statham ; *Hon. Secretary*—Mr. W. A. Jackman ; *Hon. Treasurer*—Mr. A. E. Iles ; *Committee*—Mr. F. Lace, Dr. J. O. Symes, Dr. D. C. Rayner, Mr. C. F. Walters, Professor E. Hey Groves, Dr. W. V. Wood, Dr. H. L. Ormerod ; *Library Sub-Committee. Hon. Medical Librarian*—Dr. C. K. Rudge.

It was resolved that a letter of sympathy be sent to Dr. Rudge, who was ill.

The alterations to Rules 2 and 3, passed at the last meeting, were confirmed, this completing the confirmation of the new Rules of the Society.

*November* 10th, 1926.

Dr. A. L. Flemming, President, in the Chair.

Mr. E. R. Chambers showed a case of carcinoma of the uveal tract in a woman of 55, of four months' duration. An exactly similar growth had been removed from the same site five years ago. He proposed to treat with radium.

Dr. A. D. Fraser showed specimens of endometrioma.

Mr. A. W. Adams read a paper on **"The Treatment of Congenital Club Foot."**

He pointed out the differences between this condition and the deformities consequent on paralysis, spastic or flaccid. He proposed to speak particularly of congenital talipes equinovarus, the commonest variety. The ætiology was unknown, but he inclined to the old theory put forward by Hippocrates and by Galen, that deficiency of the liquor amnii allowed pressure on the fœtus. He did not think that the theory of arrest of development could account for some of the varieties. The incidence was greatest among males ; it was said to show a familial tendency, but this was found in two cases only of his series, who were brothers. There was a tendency to associated deformities, as illustrated by one case shown, with web fingers, ankylosed elbows and wrists and other joints. The condition tended to affect both feet, and in his series this was found in half the patients. The usual deformity found at birth was shown by means of photographs and diagrams. Two factors must be recognised : rotation inwards on an antero-posterior axis brought the os calcis up under the internal malleolus, rotation inwards on a vertical axis at the mid-tarsal joint brought the forepart of the foot pointing inwards and the sole backwards. The tendons and ligaments on the inner side of the ankle-joint were shortened. The deformities resulting in later life in untreated cases were demonstrated by photographs.

In treatment two points were essential to success : (1) Early treatment, beginning at one month if possible, when the tissues were pliable and the bones relatively plastic, (2) Regular, prolonged and continuous treatment.

It was waste of time to attempt treatment on manipulative lines unless the parents realised that for success they must co-operate with the surgeon. For several weeks treatment aimed at reposition, for some months at retention, and for some years at education. The first step is to secure the reposition of the astragalus ; it is brought about by manipulation around the antero-posterior axis. The astragalus is forced in under the tibia, and the os calcis is drawn downwards and outwards under the astragalus. In addition to forced movements by the surgeon the foot is moved as far as possible in the correct position by the nurse several times a day, being held there for a few minutes. When the posterior part of the foot was rotated the anterior part was turned outwards on a vertical axis. Slight over-correction was the aim. In all cases some fibres of the deltoid ligament had to be divided, in many part of the inferior calcaneo-scaphoid ligament, and in few was tenotomy needed. A diagram showed how a roll of stockinette might be used to avoid bruising. As soon as the foot could be brought well out, it was put up in plaster for several weeks, with changes and manipulations once a fortnight. The tendency for the plaster to fall off a fat leg and foot was met by carrying it up over the knee, or by incorporating strapping. The next step consisted in tenotomy of the tendon Achillis ; without this, attempts to correct equinus would stretch the plantar ligaments. The over-correction should not be too great. A wooden sole plate was used with strapping to retain the foot in the over-corrected position. Movements and massage were continued at regular intervals ; this was the difficult time, for parents saw the foot in a good position and wished to discontinue attendances which had become wearisome. At eighteen months a malleable splint down the back of the leg and along the sole was employed, fitted with a toe-plate to resist varus. The child was encouraged to walk, but wore the splint day and night ; a small overshoe was used with the splint. The regime of massage and movements was continued long after the splint could be discarded ; by the age of three the child could learn simple exercises and so co-operate in treatment. In some cases, however, iron supports were needed for several years. In others, those which came late for treatment, cuneiform osteotomy was required. The only absolute bar to success was failure to attend the surgeon regularly for the necessary period of several years. This might be interrupted by a variety of causes—illness of parents, patients or brothers and sisters, a new baby, poverty, or, most exasperating of all, stupidity or carelessness of parents might vitiate the results of months of work. Long treatment in plaster seemed to interfere somewhat with the nutrition of the limb. Neither this nor some degree of flat foot, which was not rare, appeared to cause the patients any trouble. There was a tendency to hallux varus when the child began to walk without splints, and in some cases an inward twisting of the tibia; osteotomy of the tibia might be needed, but had not been employed for any of the cases shown.

In conclusion, Mr. Adams classified the results obtained with thirty-three feet in tabular form, to show how regularly results less than "good" were associated with irregular attendance. Eight cases were shown to illustrate the later stages of treatment, and many photographs to demonstrate the application of various forms of retention apparatus.

Professor E. W. Hey Groves congratulated Mr. Adams on his very valuable paper. He agreed that the greatest difficulty in treatment was to secure the efficient co-operation of parents. He thought that sufficient emphasis had not been given to the correction of the malposition of the os calcis. He had found that reposition in a small fat foot was not easy, but thought failures were often due to this part of the deformity not having been corrected. Where the foot looks inwards he advised osteotomy of the tibia. Tarsectomy gave excellent immediate results as regards reposition, but the after-treatment needed to be just as careful and prolonged, and the final result was not so good as with manipulative methods. He particularly admired the sole piece used by Mr. Adams.

Mr. H. Chitty agreed that the displacement of the os calcis was the most difficult part of the deformity to reduce. He found plaster splints very difficult to retain in position. He used a small internal malleable splint. In late cases he took out a wedge on the outer side, to correct the heel, and found the remaining correction easy.

Mr. E. Watson-Williams noticed that the first case shown and several of the photographs had the second or third toe pushed up out of line, so as to lie on the dorsum of their neighbours. He asked whether this disappeared of itself later, what was required to correct it if not, and whether it was connected with the tendency to hallux adductus.

Mr. W. A. Jackman admired Mr. Adams's splints, and used them regularly. He had obtained very good results in seven cases out of eight with splints, not using plaster; he asked Mr. Adams's advice as to treatment in an eighth case, $2\frac{1}{2}$ years old, that had not improved in spite of the most careful and intensive treatment since six weeks old. All his cases were bilateral.

The PRESIDENT asked whether the almoner's department had been used to follow up the cases. He added that the wealthy failed to get the best treatment, as well as the very poor; they were inclined to go from one surgeon to another, and in consequence continuous treatment by one method was lost. He believed this deformity was related to defective heredity, such was relatively common in remote and isolated districts, and which showed itself in mental as well as physical abnormalities.

MR. ADAMS in reply said he recognised that the posterior deformity was the most difficult to treat, and referred to the passages and diagrams in his paper which dealt with this. This correction can be made at the same time as the abduction, and it is not difficult to overdo it. In favour of plaster instead of splints over at the early stage, he thought splints needed daily attendance. He admitted that the following of cases would be easier if there were an officer specially for this service. Great efforts were often needed to keep some parents attending regularly.

E. WATSON-WILLIAMS.
W. A. JACKMAN, *Hon. Sec.*

